# Emw1/TTC27 is a chaperone required for folding of the eukaryotic elongation factor 2

**DOI:** 10.1007/s00018-026-06154-9

**Published:** 2026-03-10

**Authors:** Mengqi Yang, Ruixin Li, Anna I. Mikolajczak, Vanessa A. Wright, Mahnoor Hassan, Cara K. Vaughan, Thomas A. K. Prescott, Jennifer A. Heritz, Mehdi Mollapour, Barry Panaretou

**Affiliations:** 1 Institute of Pharmaceutical Science, School of Cancer and Pharmaceutical Sciences, King’s College London, Franklin-Wilkins Building, 150 Stamford Street, London, SE1 9NH UK; 2 Current Address RNA Therapeutics Institute UMass Chan Medical School, Albert Sherman Center (AS4-2046), 368 Plantation Street, Worcester, MA 01605 USA; 3 Current Address Experimental Histopathology, The Francis Crick Institute, 1 Midland Road, London, NW1 1AT UK; 4 Department of Molecular Biosciences, University of Texas at Austin, Austin, TX USA; 5 Royal Botanic Gardens, Kew, Richmond TW9 3AB, Surrey, UK; 6 Department of Urology, SUNY Upstate Medical University, Syracuse, NY 13210 USA; 7 Department of Biochemistry and Molecular Biology, SUNY Upstate Medical University, Syracuse, NY 13210 USA; 8 Upstate Cancer Centre, SUNY Upstate Medical University Syracuse, Syracuse, NY 13210 USA

**Keywords:** Chaperone, Protein folding, Protein stability

## Abstract

Eukaryotic elongation factor 2 (eEF2) requires extensive chaperone support due to its complex multi-domain structure and high abundance. However, the full complement of chaperones involved in its folding remains elusive. Here, we identify a protein that bears TetraTriCopeptide repeats, Emw1/TTC27, as a critical chaperone for eEF2 folding. This essential protein has been conserved throughout the eukaryotic lineage. Using the budding yeast *Saccharomyces cerevisiae*, we show that Emw1/TTC27 physically interacts with eEF2, and levels of the elongation factor decline when activity of Emw1/TTC27 is limited. Emw1/TTC27 facilitates folding during eEF2 synthesis rather than stabilizing pre-folded eEF2. This role parallels that of Hgh1, another known eEF2 chaperone, with evidence suggesting that Emw1/TTC27 and Hgh1 cooperate, as dual impairment of these proteins severely restricts yeast growth. In the *hgh1Δ* background expressing a hypomorphic allele of *EMW1*, additional deletion of one of the two eEF2 paralogs is lethal, underscoring the essential role of Emw1/TTC27 in preventing toxic folding intermediates during eEF2 biogenesis. Taken together, our data suggest that Emw1/TTC27 is a critical addition to the eEF2 chaperone network, acting co-translationally with other chaperones to ensure eEF2 folding and maintain proteostasis.

**Supplementary Information:**

The online version contains supplementary material available at 10.1007/s00018-026-06154-9.

## Introduction

Most proteins are composed of multiple domains, however intra- and inter-domain misfolding can occur to yield non-native structures which will compromise cellular proteostasis, especially if this occurs during the synthesis of abundant proteins. Interaction with the ribosome and with molecular chaperones are essential for synthesis of correctly folded protein [1]. The synthesis and folding of translation elongation factor G (EF-G) in prokaryotes has been used as a paradigm for generating mechanistic insight regarding how multi-domain proteins fold, and these studies have been extended to its orthologue eEF2 (eukaryotic Elongation Factor 2), specifically the identical *Saccharomyces cerevisiae* orthologues encoded by *EFT1* and *EFT2* [2–4]. This protein drives the GTP-dependent translocation of the nascent protein chain from the A-site to the P-site of the ribosome, and is composed of five non homologous domains: two successive GTPase domains (G and G’) and domain II are followed by domains III-V at the C terminal end [5].

Recent work using the *S.cerevisae* model revealed that eukaryotic cells devote a significant portion of their chaperone armory, to the folding of eEF2. The highly conserved chaperone Hgh1 recognises a non-native conformation of domain III of eEF2, such a conformation would only be present during eEF2 biogenesis. Accordingly the chaperone activity of Hgh1 may occur co-translationally, and is thought to prohibit aberrant interaction between the domains of eEF2 that flank domain III [4]. Additionally, Hgh1 recruits the chaperonin TRiC a hexadecameric double-ring complex which forms a cavity that can accommodate substrates or domains thereof that do not exceed 70 kDa. TRiC interacts with domains III-V of eEF2, with encapsulation of these domains in the TRiC chamber facilitating their folding [4]. This is likely to occur in a step-wise fashion, as indicated by the encapsulation of successive C terminal domains of the splicing factor of Snu114, a structural homolog of eEF2 [6]. Folding of eEF2 is also facilitated by the Hsp90 chaperosome, as evidenced by the decline in levels of eEF2 when yeast cells are incubated in the presence of the Hsp90 inhibitors macbecin or radicicol [3, 4]. Hsp90 is usually shunted towards folding a specific client by a co-chaperone, in this case the co-chaperone is Cns1 [3]. This is an essential protein that bears three tetratricopeptide repeat (TPR) motifs at its N terminus. TPR domains mediate protein–protein interactions, and it is this domain of Cns1 that binds Hsp90 [7]. Additionally, proteome wide interaction screens implicate members of the Hsp70 family (Ssa1, Ssa2, Ssb1 and Ssb2), and the Hsp70 co-chaperone Ydj1 in the folding of eEF2 [8–12].

We identified the product of *EMW1* as another potential chaperone that could fold eEF2 by examining the *S.cerevisiae* protein-protein interaction data set generated via a protein complementation assay (PCA) where bait and prey proteins are fused to the N and C terminal domains of murine dihydrofolate reductase (mDHFR) [13]. We focused on this data set as opposed to any of the other reported proteome-wide studies, as a screen based on a cytosolic PCA would identify proteins that interact both co- and post- translationally. *EMW1* (Essential for Maintenance of the cell Wall), is the yeast orthologue of human TTC27 (TeTratriCopeptide repeat protein 27), a conserved eukaryotic gene encoding a 102.3 kDa protein that bears six tetratricopeptide repeats towards its C-terminus [14]. Despite being an essential protein in *S.cerevisiae*, the cellular role of Emw1 remains poorly understood with only two publications attempting to reveal its function. One revealed its role in maintenance of cell wall integrity in *S.cerevisiae* [14]; another highlighted its vital function for cell fate determination in *Caenorhabditis elegans* [15]*.* Although a temperature sensitive *S.cerevisiae* mutant (*emw1*^*ts*^) loses cell wall integrity at non-permissive temperatures, the primary cellular role of the protein is unlikely to be directly associated with cell wall synthesis since the corresponding gene is found in all eukaryotes including humans, and the cell wall is not a feature of all eukaryotic cells. Cell wall integrity signalling pathways are not compromised in *emw1*^*ts*^ mutants and Emw1 does not play a direct role in synthesis of cell wall polymers like chitin [14, 16]. The robust cell wall phenotype that is displayed by *emw1*^*ts*^ mutants reflects how important the cell wall is to yeast, which means disturbing a diverse array of cellular activities may lead to a cell wall defect. This turns out to be the case as defects in cohesin [17], the nuclear exosome [18], and the mitotic exit network [19], all lead to cell wall defects. A search of the databases failed to identify proteins that share significant sequence identity with the N terminal and C terminal domains that lie either side of the Emw1/TTC27 TPR domain. In this study we show that Emw1/TTC27 is a chaperone with one client identified thus far, eEF2, the eukaryotic elongation factor 2. Levels of eEF2 decline when Emw1/TTC27 function is compromised, a decline which is exacerbated in a background lacking Hgh1, a known eEF2 chaperone. Levels of eEF2 synthesized prior to loss of Emw1/TTC27 function remain the same for hours, suggesting Emw1/Ttc27 is required for folding during synthesis as opposed to being required for maintaining stability of already-folded eEF2.

## Results

### Emw/TTC27 binds to the translational GTPases eEF2 and eEF1A

Individual pair-wise interactions identified by via the PCA screen can be repeated because yeast strains expressing all proteins as either fusions to the N -terminal domain of murine DHFR or the C-terminal domain of DHFR, are commercially available (Horizon Discovery). The screen uses a mutant murine DHFR that is 10,000 fold less sensitive to methotrexate than the wild-type DHFR native to yeast. Methotrexate inhibition of yeast DHFR is complemented by activity of re-constituted murine DHFR. Furthermore, the library was constructed by targeted integration of DNA encoding the DHFR domains at the 3’ ends of yeast ORFs, so the expression of the fusion genes occurs via the native promoters so interactions are detected using endogenous levels of protein [13].

**Fig. 1:**
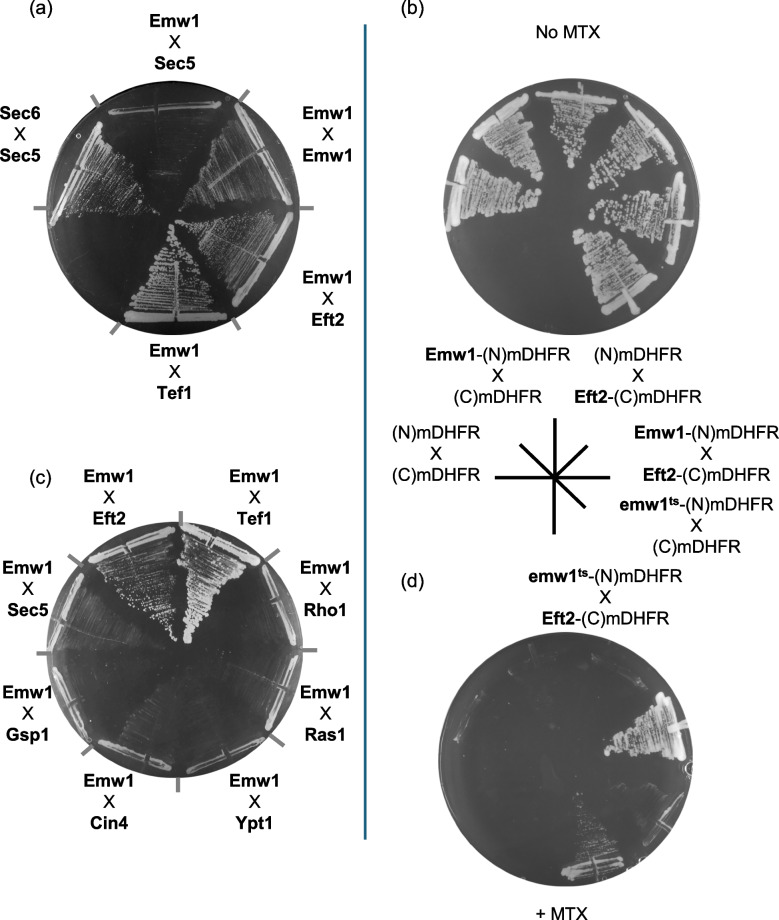
Physical interactions between proteins, revealed using an in vivo protein complementation assay (PCA). (**a** and **c**) *S. cerevisiae* diploids expressing bait composed of *EMW1* tagged at the 3’ end with DNA encoding the N -terminal domain of murine Dihydrofolate reductase (N)mDHFR, and the prey open reading frames indicated, tagged at the 3’ end with DNA encoding the C -terminal domain of murine Dihydrofolate reductase, (C)mDHFR. Bait and prey are expressed at native levels because fusions were generated via tagging the genomic copies of the ORFs. Cells were incubated at 30 °C for 2 days on SD media lacking methionine and lysine (which only permits growth of diploids) containing 100 μg/ml methotrexate. (**b**) *S. cerevisiae* bearing episomal plasmids expressing the indicated pair-wise combinations of (N) mDHFR, (C)mDHFR, Emw1-(N)mDHFR, emw1^ts^-(N)mDHFR and Eft2-(C)mDHFR, all under the control of the constitutively active promoter of *ADH1*. Cells were incubated at 30 °C for 2 days on SD media lacking leucine and uracil (No MTX) and the same media also containing 100μg/ml methotrexate (+ MTX)

As expected, we detected the interaction between Emw1/TTC27 and yeast eEF2 (encoded by *EFT2* in yeast) that was initially reported by the screen performed by Tarassov and co-workers [13] (Fig. 1a). We used the well-established interaction between Sec6 and Sec5 as a positive control [20] (Fig. 1a). A *bona fide* negative control was not available as strains expressing the N terminal domain or C terminal domain of DHFR domain only, are not available because the library was constructed by integrating cassettes into the genome. An interaction between Emw1 and Sec5 has never been reported by any of the numerous proteome-wide interaction screens including those exploiting the yeast two hybrid trap and tandem affinity purification, so we used this as a negative control. As expected, we did not detect an interaction between Emw1 and Sec5 using the corresponding PCA strains (Fig. 1a). To mitigate against the possibility of a false positive in this case, we constructed centromeric (single copy number) recombinant vectors that expressed the N -terminal domain of mDHFR (N mDHFR) only, or an Emw1-(N mDHFR) fusion, the C -terminal domain of mDHFR (C mDHFR) only or an Eft2-(C mDHFR) fusion. All genes were expressed from the constitutively active *ADH2* promoter. As expected, the negative controls, namely Emw1-(N mDHFR) x (C mDHFR) and (N mDHFR) x Eft2-(C mDHFR) did not grow on methotrexate (Fig. 1d). Accordingly, the interaction between Emw1 and Eft2, is not a false positive and is likely to be physiologically relevant. Proteome-wide data sets using affinity tags and co-immunoprecipitation techniques indicate that tagged Emw1/TTC27 also pulls down the untagged Emw1/TTC27 [21]. This suggests interactions occur between Emw1 polypeptides, forming an oligomer. The yeast diploid expressing Emw1-(N mDHFR) and Emw1-(C mDHFR) fusions grew on methotrexate, suggesting that Emw1 monomers can interact (Fig. 1a).

eEF2 is a translational GTPase (trGTPase), these GTPases share similar structural elements extending beyond the nucleotide binding domains [22]. eEF1A is a trGTPase that acts prior to eEF2, delivering elongator aminoacyl-tRNAs to the A site. In yeast, eEF1A is encoded by two genes *TEF1* and *TEF2* (expressing identical proteins). We tested Tef1, and found that it physically interacts with Emw1/TTC27 (Fig. 1a). The interaction between Emw1/TTC27 and the GTPases eEF2 and eEF1A, raised the possibility that Emw1/TTC27 may be a generic chaperone for GTPases. Therefore, we tested for interaction with the small GTPases, monomeric GTPases classified by structure into five families, Ras, Rho, Rab, Sar1/Arf, and Ran [23]. We tested one member of each of the five families (Rho1, Ras1, Ypt1, Arf1 and Gsp1), and none of them interacted with Emw1/TTC27 (Fig. 1c).

### Steady state levels of eEF2 are reduced when function of Emw1/TTC27 is compromised

**Fig. 2:**
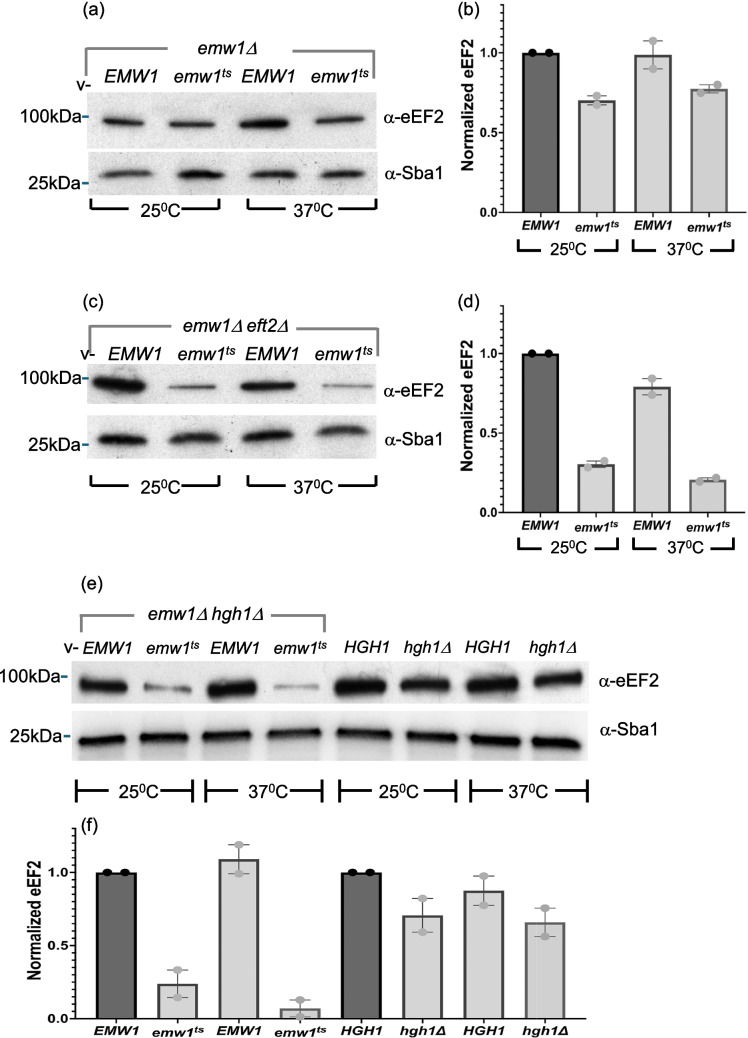
(**a**-**f**) Impaired Emw1 function leads to a decline in levels of eukaryotic Elongation Factor 2 (eEF2). emw1Δ (**a**, **b**), *emw1Δeft2Δ*. (**c**, **d**), *emw1Δhgh1Δ* (**e**, **f**), bearing a centromeric vector (v-) expressing either wild type *EMW1* or *emw1*^*ts*^ from the *EMW1* promoter. Cultures were incubated in YPD to exponential phase at 25 °C, split into two equal aliquots which were either i) maintained at 25 °C or ii) shifted to 37 °C for four hours. Extracted proteins were resolved by SDS-PAGE and Western blots were probed with anti-eEF2 antisera or anti-Sba1 antisera (as loading control). Lanes were loaded with 4μg protein; molecular weight markers are indicated on the left. The last four lanes of e and f are extracts from wild type and *hgh1Δ* cells to observe the difference between the *emw1*^*ts*^*hgh1Δ *double mutant and the single *hgh1Δ* mutant. Densitometry analysis is presented for all Western blots, where eEF2 levels are indicated on bar charts (Fig. 2b,d and f); averages with SD from two independent experiments are shown, as a proportion of wild-type control (i.e. includes a correction based on the ratio between eEF2 and the loading control in each lane)

**Fig. 3:**
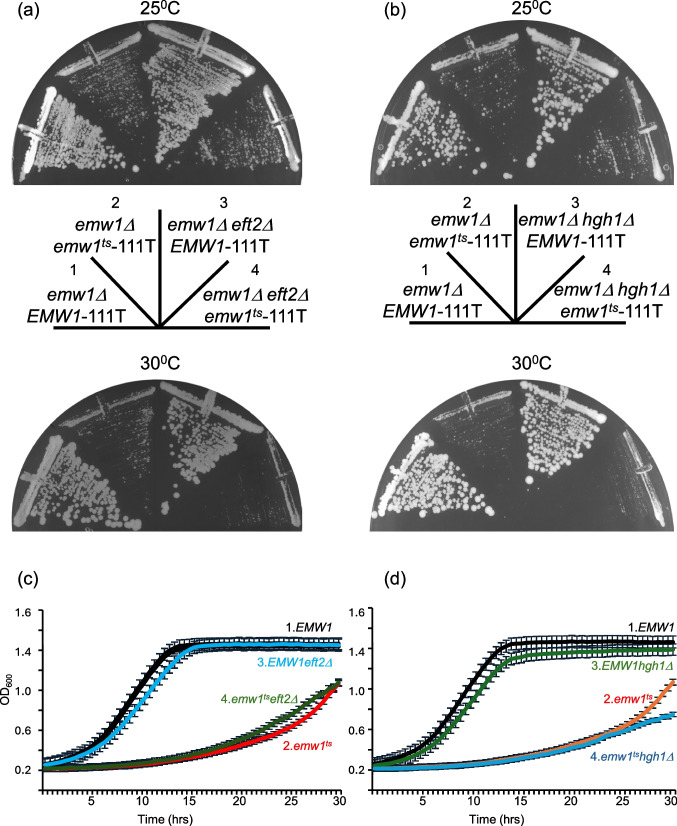
Impaired Emw1 function limits cell growth. (**a**) *emw1Δ*, *emw1Δeft2Δ* and (**b**) *emw1Δhgh1Δ* strains bearing *EMW1* on a centromeric *URA3* vector were transformed with a centromeric *LEU2* vector bearing wild-type *EMW1* or *emw1*^*ts*^. Transformants were subsequently incubated on SD with 5-fluoroorotic acid for 3 days at 25^0^Cor 30^0^C (5-fluoroorotic acid selects for cells that have lost the centromeric *URA3* vector). (**c**) and (**d**) are growth curves for the same strains in YPD at 250^C^.

Protein–protein interactions can either influence function of a polypeptide or levels of a polypeptide per se. *EMW1* is an essential gene and our prior work investigated the consequences of loss of Emw1 function using five temperature sensitive alleles that were generated via random mutagenesis [14]. The work described below uses allele *emw1-1*^*ts*^ which bears the following amino acid substitutions: L10S, V422G, and Y435C (*this allele will be referred to as emw1*^*ts*^* from now on*). An isogenic pair of strains were used in which the genomic copy of *EMW1* is deleted, but cell viability is maintained by a copy of either the wild-type *EMW1* or *emw1*^*ts*^ on a centromeric vector, where expression of *EMW1* or *emw1*^*ts*^ is driven by the native *EMW1* promoter. Wild-type and mutant cells were shifted to the non-permissive temperature (37^0^C) for a duration sufficient to compromise function in the *emw1*^*ts*^ cells (as evidenced by an arrest in growth) but prior to cell death, which started to occur after 4 h at 37^0^C. For this reason we extracted protein after 4 h at non-permissive temperature to ensure that protein measurements reflected viable cell conditions rather than the additional secondary effects of cell death. There is a moderate, yet reproducible, loss of eEF2 at both permissive and non-permissive temperature, when levels of this protein were compared between the *EMW1* and *emw1*^*ts*^ cells (a ca. 30% decline in levels at 25^0^C, Figs. 2a-b). The *emw1*^*ts*^ mutant is viable at permissive temperature, but because the mutation is hypomorphic, Emw1 activity is still partly lost. This is reflected by reduced eEF2 levels (Figs. 2a-b) and poor growth (Fig. 3a. compare sectors 2 and 1 on solid media maintained at 25^0^C, with a clearer effect visible when using a more sensitive assay, Fig. 3c compare growth curves 2 and 1).

eEF2 is one of the most abundant proteins in *S.cerevisiae* [24]. It is encoded by two genes, *EFT1* and *EFT2*, both genes encode identical proteins, specifically *EFT1* produces 30%, and *EFT2* produces 70% of the total transcript [25]. Two copies are present owing to the ancient duplication of the *S.cerevisiae* genome [26]. Though the *eft1Δeft2Δ* double delete is inviable, the single deletes can grow [27]. Therefore, eEF2 levels were assessed again, this time using strains in which *EFT2* had been deleted, so the only source of eEF2 is *EFT1*. In this case, there was a dramatic loss of eEF2 levels. In the *emw1*^*ts*^ background there was a ca.70% decline at permissive temperature compared to wild-type *EMW1*, and a ca. 80% decline by four hours after an upshift to the non-permissive temperature (Fig. 2c and d). The *emw1*^*ts*^ mutant does not grow at 37^0^C, so we incubated cells at a semi-nonpermissive temperature (30^0^C) to investigate effects on growth rate. Not surprisingly, at this temperature growth is slower in *emw1*^*ts*^*eft2Δ* compared to *emw1*^*ts*^ (Fig. 3a, compare sectors 4 and 2 at 30^0^C).

### The additive effect on eEF2 levels, in cells that lack Hgh1

*HGH1* is a chaperone required during eEF2 biogenesis, it not essential in yeast but it has been reported that deletion of this gene leads to a modest ca. 35% reduction in eEF2 levels [4], and we report a similar outcome with a ca. 30% reduction in eEF2 (compare 5th and 6th lanes in Fig. 2e, and corresponding quantification in Fig. 2f). If Emw1/TTC27 is a chaperone required for eEF2 folding, then we would expect to see an additive effect on eEF2 levels if the activity of both Emw1/TTC27 and Hgh1 is compromised. This turns out to be the case, a dramatic decline in eEF2 levels is evident in the *hgh1Δ* background when Emw1 function is compromised. There was a ca.75% decline at permissive temperature compared to wild-type *EMW1*, and a ca. 80% decline by four hours after an upshift to the non-permissive temperature (Fig. 2e and f). Notably, this was evident in a background expressing both genes (*EFT1* and *EFT2*) that encode eEF2. Not surprisingly, growth is slower in *emw1*^*ts*^*hgh1Δ* compared to *emw1*^*ts*^ at 30^0^C, a semi-nonpermissive temperature for the *emw1*^*ts*^ mutant (Fig. 3b compare sector 4 with sector 2 at 30^0^C).

**Fig. 4:**
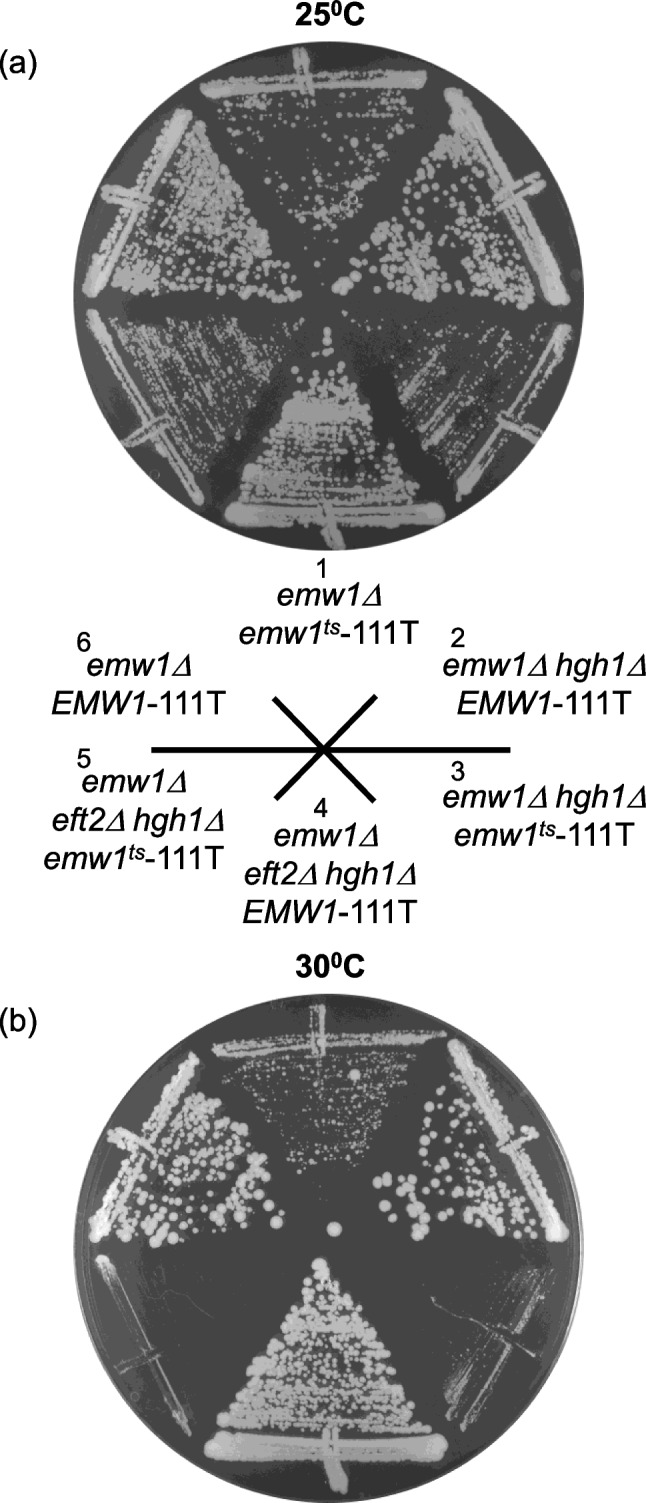
Lack of Hgh1, and impaired function of Emw1, is synthetic lethal in an *eft2Δ*. *emw1Δ*, *emw1Δhgh1Δ* and *emw1Δeft2Δhgh1Δ* strains bearing *EMW1* on a centromeric *URA3* vector were transformed with a centromeric *LEU2* vector (111 T) bearing wild-type *EMW1* or *emw1*^*ts*^. Transformants were subsequently incubated on SD with 5-fluoroorotic acid for 3 days at 25^0^C or 30.^0^C (5-fluoroorotic acid selects for cells that have lost the centromeric *URA3* vector)

The genetic relationship between Emw1, Hgh1 and eEF2 was explored further by generating an *emw1*^*ts*^*hgh1Δ eft2Δ* background. An additive growth defect at 30^0^C is clear i.e. *emw1*^*ts*^ grows faster than *emw1*^*ts*^*hgh1Δ* which in turn grows faster than *emw1*^*ts*^*hgh1Δ eft2Δ*, indeed this latter triple mutant strain is synthetic lethal at 30^0^C (Fig. 4b, compare sectors 1, 3 and 5, at 30^0^C).

### Emw1/TTC27 is required for the folding of eEF2 during synthesis

**Fig. 5:**
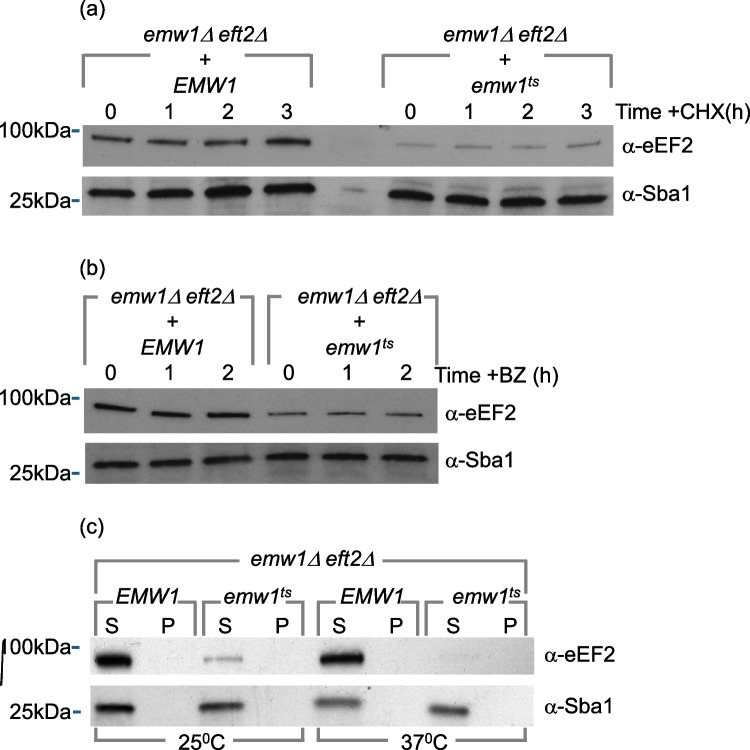
(**a**) Stability of mature eEF2 when Emw1 function is compromised. *emw1Δeft2Δ *bearing a centromeric vector (v) expressing either wild type *EMW1* or *emw1*^*ts*^ from the *EMW1* promoter were grown in YPD at 25 °C to exponential phase and were treated with the protein synthesis inhibitor cyclohexiimide (CHX, 0.1 mM). After the indicated time points, protein was extracted and resolved by SDS-PAGE and Western blots were probed with anti-eEF2 antisera or anti-Sba1 antisera (as loading control). Lanes were loaded with 4μg protein; molecular weight markers are indicated on the left. (**b**) Levels of eEF2 when action of the 26S proteosome is inhibited. The same analysis described in (a) but using the 26S proteasome inhibitor Bortezomib (BZ, 0.1 mM) instead of cycloheximide. (**c**) Misfolded eEF2 does not accumulate as insoluble aggregates when Emw1 function is compromised. *emw1Δeft2Δ* bearing a centromeric vector (v) expressing either wild type *EMW1* or *emw1*^*ts*^ from the *EMW1* promoter were grown in YPD at 25 °C to exponential phase, split into two equal aliquots which were either i) maintained at 25 °C or ii) shifted to 37 °C for four hours. Lysates were fractionated by centrifugation. eEF2 protein and Sba1 loading control were assessed in soluble (S), and pellet fractions (P).

Molecular chaperones can act co- or post-translationally. Chase experiments using cycloheximide did not impact the turnover of eEF2 in *eft2Δemw1*^*ts*^ cells at 25 °C (Fig. 5a). This suggests that Emw1/TTC27 is required for the folding of eEF2 during synthesis, not for the maintenance of already folded eEF2. This is similar to the role reported for Hgh1 [4], suggesting that loss of Emw1 action leads to loss of stability during eEF2 synthesis.

The level of eEF2 in the same genetic background in the presence of bortezomib, the 26S proteasome inhibitor, did not change when cells were incubated for 2 h with this drug (Fig. 5b), indicating that misfolded eEF2 is being degraded in a proteasome-independent manner.

Loss of chaperone activity leads to loss of stability, and misfolding of chaperone substrates resulting in either i) accumulation of insoluble aggregates, as is the case for actin when levels of the TRiC chaperone are reduced via knockdown [28] or ii) rapid degradation of the misfolded polypeptides, as is the case for the kinases Raf1 and c-ErbB2, when Hsp90 action is inhibited [29, 30]. We have fractionated extracts to separate the soluble from insoluble protein, and we find that loss of Emw1/TTC27 function leads to rapid clearance of eEF2 as opposed to build-up of eEF2 aggregates (Fig. 5c).

### Mutation of Emw1/TTC27 renders cells hypersensitive to an inhibitor of eEF2

**Fig. 6:**
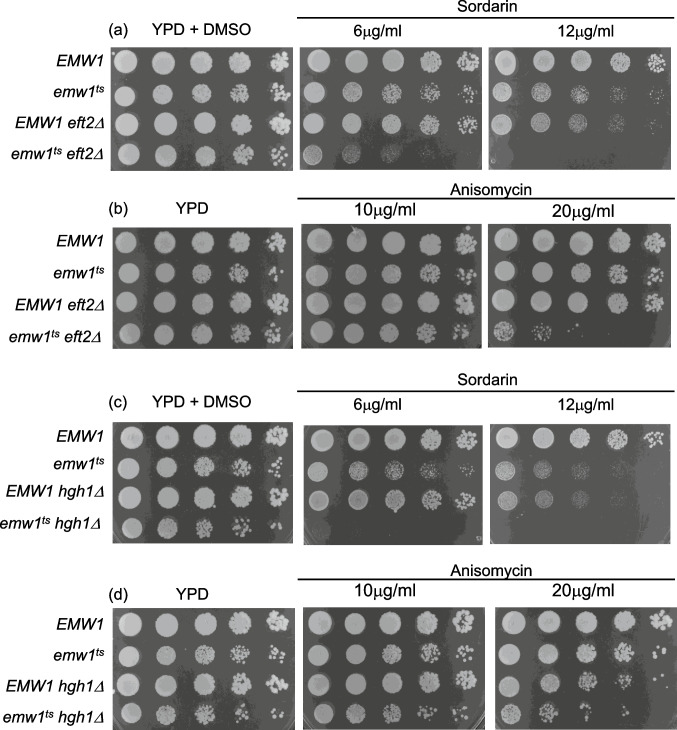
Limiting the action of Emw1 renders cells hypersensitive to sordarin, an eEF2 inhibitor. Strains indicated were grown on YPD at 25 °C to exponential phase and diluted to equal cell density. Five-fold serial dilutions were spotted across YPD without any drug (YPD) or DMSO only (using the volume of DMSO corresponding to that used to add the highest drug concentration), or containing sordarin or anisomycin at the concentrations indicated. Cultures were incubated at 25 °C for 3 days

If Emw1 plays a role in the function of eEF2, then compromise of Emw1 function would render cells hypersensitive to drugs that inhibit the elongation phase of translation. This turns out to be the case, as *emw1*^*ts*^ was moderately sensitive to sordarin at 25 °C, but *emw1*^*ts*^*eft2Δ* was hypersensitive to this drug (Fig. 6a). The target of sordarin is eEF2 itself, binding to the interface of domains III IV and V of eEF2 [5]. Less sensitivity was displayed towards anisomycin (Fig. 6b), another translation elongation inhibitor, but that is not surprising as its target is not eEF2 but the peptidyl transferase activity associated with the 80S ribosomal subunit [31]. The *emw1*^*ts*^*eft2Δ* strain was hypersensitive to sordarin because of two reasons. Firstly, levels of the drug target are reduced as one of the genes encoding eEF2 was deleted (*eft2Δ*) Secondly, the eEF2 that remains in the *eft2Δ* background was de-stabilized by loss of Emw1 action (*emw1*^*ts*^). It is notable that an *emw1*^*ts*^*hgh1Δ* strain was also hypersensitive to sordarin (Fig. 6c), indicating that compromising the action of two eEF2 chaperones (Hgh1 and Emw1) leads to a significant reduction in functional eEF2 levels even in a strain where both genes that express eEF2 are intact. As was reported for *emw1*^*ts*^*eft2Δ* (Fig. 6a and b), the *emw1*^*ts*^*hgh1Δ* sensitivity to sordarin is greater than sensitivity to anisomycin (Fig. 6c and d) because the anisomycin target is not eEF2 but the peptidyl transferase activity associated with the 80S ribosomal subunit [31].

### Assessing interaction of eEF2 domains with Emw1/TTC27

**Fig. 7:**
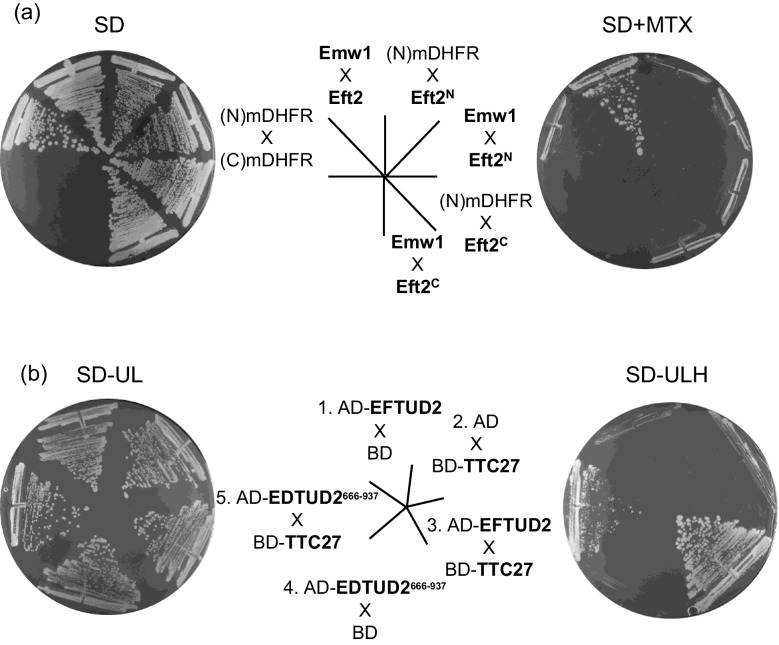
The N -terminal GTPase domain of eEF2 does not interact with Emw1. (**a**) in vivo protein complementation assay (PCA). *S. cerevisiae* diploids bearing episomal plasmids expressing the indicated pair-wise combinations of: Emw1-(C)mDHFR, Eft2^N^-(C)mDHFR, Eft2^C^-(C)mDHFR. Eft2^N^ is the N-terminal GTPase module (residues 1–482), Eft2^C^ is the C-terminal module (residues 484–842) comprising domains III, IV, and V. (N)mDHFR and (C)mDHFR are also expressed as controls. In all cases, expression is driven by the *ADH1* promoter. Cells were incubated at 30 °C for 2 days on SD media lacking leucine and uracil (SD) and the same media also containing 100μg/ml methotrexate (+ MTX). (**c**) Physical interaction in vivo between TTC27 and the C terminal end of EFTUD2. Two-hybrid interaction in diploids expressing a Gal4 DNA binding domain (BD)-TTC27 fusion and the Gal4 activation domain (AD) fusions indicated. Cells were incubated at 30^0^C on SD media lacking uracil, leucine (SD -U,L) and SD -UL lacking histidine (SD -ULH). Interaction between AD- and BD-fusions is scored by growth on media lacking histidine, as this leads to expression of *HIS3* driven by the *GAL1* promoter.Accession number for TTC27 isoform NP_060205.3; accession number for EFTUD2 isoform NP_004238.3

**Fig. 8:**
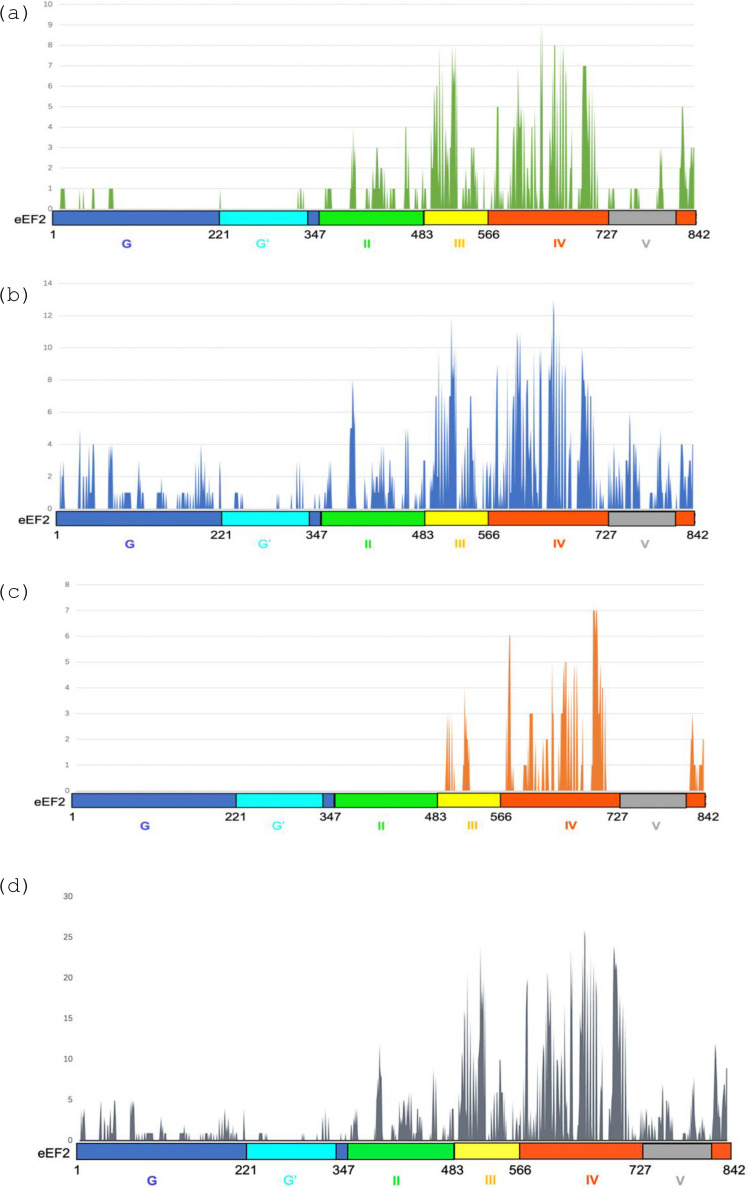
Docking prediction of Emw1 binding to eEF2. The x-ray structure of yeast *S.cerevisiae* eEF2 (PDB:1N0V) and predicted structure of yeast *S.cerevisiae* Emw1 (AlphaFoldDB: P42842) were docked by ClusPro server [32] and the interface residues were analyzed in PYMOL. Domains of eEF2 are indicated. High scoring models are presented from the (**a**) balanced, (**b**) electrostatic and (**c**) hydrophobic-favoured modes and (**d**) a composite of all three. The docking models selected were those with cluster sizes > 10 and weighted score more negative than −750 (an energy-based score, assigned to a representative structure of each cluster, based on a weighted sum of physical interaction terms). This combination was selected because a more negative weighted score indicates a more favourable interaction, and a cluster with many members indicates that many independent docking attempts converged on a similar solution, suggesting a more reliable binding mode [67, 68]

We attempted to determine which domain of eEF2 interacts with Emw1/TTC27. To begin with, we divided yeast Eft2 (encoding the yeast eEF2 orthologue) into two regions, the N domain GTPase (residues 1–482, eEF2^N^) consisting of the G, G’ and II domains and the C terminal region composed of domains III, IV and V (residues 483–842, eEF2^C^; see schematic diagrams in Fig. 8). We generated recombinants that would express the corresponding fragments fused to the C terminal domain of mDHFR, with the fusion gene under the control of the strong constitutive promoter of *ADH2*. We tested for interaction with a protein composed of Emw1 fused to the N domain of mDHFR. Neither of the eEF2 fragments interacted with Emw1/TTC27, in contrast to the strong interaction between Emw1/TTC27 and full length eEF2 (Fig. 7a). It has been reported that eEF2^N^ can be expressed in a soluble form in yeast, but eEF2^C^ is insoluble [4]. Our own data infers that Emw1 does not interact with eEF2^N^ (Fig. 7a). An interaction with eEF2^C^ might occur, but only in the context of full length eEF2, as the eEF2^C^ domain expressed by itself is not soluble. In agreement with this, the ClusPro docking server predicted a greater likelihood of interaction between eEF2^C^ and Emw1/TTC27, in comparison to interaction between eEF2^N^ and Emw1/TTC27 (Fig. 8a-d) [32]. The outcome of a screen for proteins that interact with the human orthologue of Emw1 (TTC27) supports the possibility of an interaction between eEF2^C^ and Emw1/TTC27. A yeast-2-hybrid screen was performed using human TTC27 as bait and the Matchmaker cDNA prey fusion protein library from human leukocytes. The screen identified four interaction partners, one of these was the C terminal domain of the splicing factor EFTUD2, namely a fragment composed of the last 272 amino acids of the protein (Fig. 7b, sector 5). EFTUD2, the orthologue of *S.cerevisiae* Snu114, is a closely related paralog of eEF2, the proteins sharing the same arrangement of domains, namely two successive GTPase domains (G and G’) and domain II, followed by domains III-V at the C terminal end [6] (supplementary Fig. S1a). The 272 amino acid C terminal fragment of EFTUD2 is composed of domains IV and V, displaying a high degree of similarity to the equivalent domains in eEF2 (supplementary Fig. S1b). The interaction between this domain of EFTUD2 and TTC27 is not an artefact related to polypeptide truncation, because we generated a recombinant that expressed Gal4 Activation domain fused to full length EFTUD2, and showed that it interacts with the Gal4 DNA Binding domain-TTC27 fusion that we used as bait for the two hybrid screen (Fig. 7b, sector 3).

**Fig. 9:**
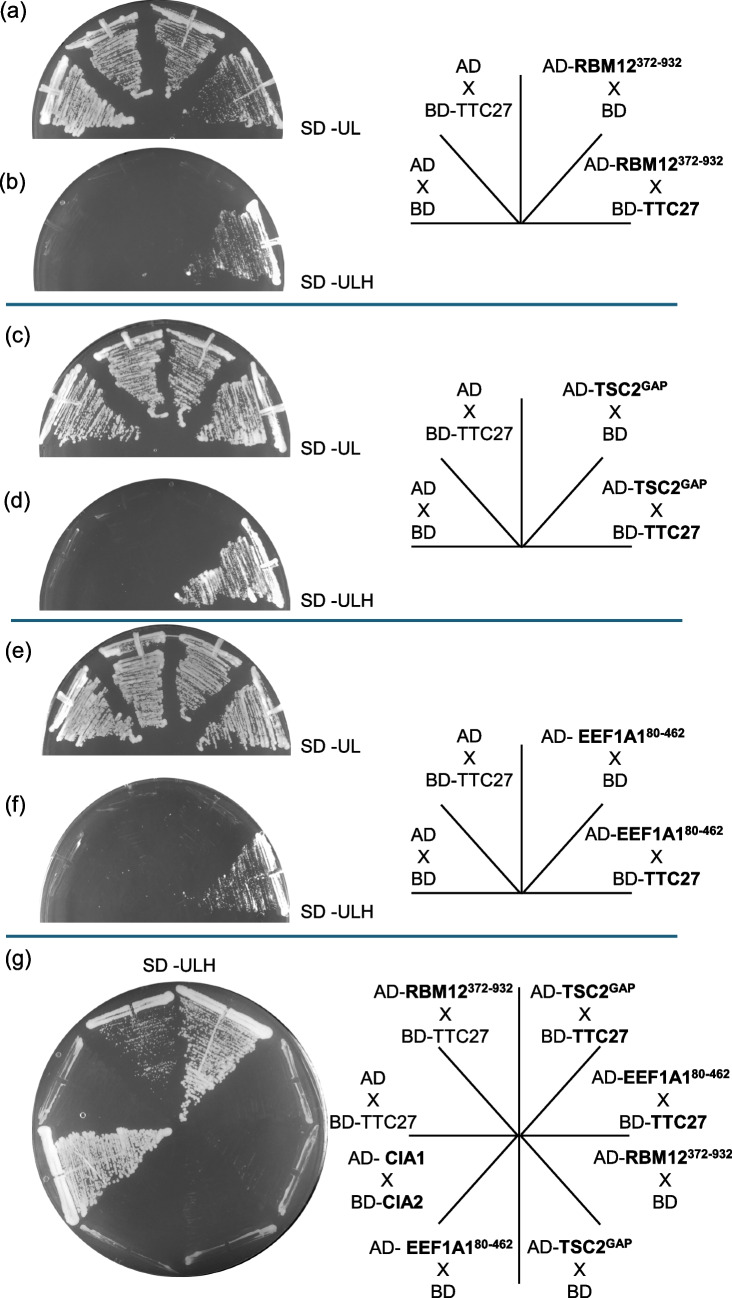
Physical interaction between TTC27 and RBM12^372−932^, TSC2^GAP^ and EEF1A1^80−462^. Two-hybrid interaction in diploids expressing a Gal4 DNA binding domain (BD)-TTC27 fusion and the Gal4 activation domain (AD) fusions indicated. Cells were incubated at 30.^0^C on SD media lacking uracil, leucine (SD -U,L) (**a**), (**c**) and (**e**) or SD -ULH (lacking histidine) (**b**), (**d**), and (f). Interaction between AD- and BD-fusions is scored by growth on media lacking histidine, as this leads to expression of *HIS3* driven by the *GAL1* promoter. Accession number for TTC27 isoform NP_060205.3. An inhibitor of the His3 reporter product (3-amino triazole, at 1 mM) is included (g), permitting comparison of strength of interaction between the AD fusions indicated and (BD)-TTC27. The strong interaction between Cia1 and Cia2 is included as positive control [69]

The two hybrid screen also revealed interaction between TTC27 and residues 372–932 of RNA binding motif containing 12, RBM12 (referred to as RBM12^372–932^); residues 1490-1763 of the tumor suppressor tuberin (TSC2) which turns out to be the GTPase activating domain of this protein (referred to as TSC2^GAP^) and residues 80–462 of eukaryotic translation elongation factor 1 A, eEF1A1, referred to as eEF1A1^80−462^ (Fig. 9b, d and f). Use of 3-aminotriazole (an inhibitor of the His3 reporter for the two hybrid trap) indicates that interaction between TTC27 and TSC2^GAP^ is stronger than that between TTC27 and eEF1A1^80–462^ and between TTC27 and RBM12^372−932^ (Fig. 9g). eEF1A1 is one of the eukaryotic elongation factor 1 alpha isoforms, the yeast orthologue of this protein is encoded by *TEF1*.

### Assessing levels of eEF1A when Emw1/TTC27 action is compromised

We revealed a physical interaction between Emw1/Ttc27 and both translation type GTPases eEF2 and eEF1A (the latter encoded by genes *TEF1* and *TEF2* in *S.cerevisiae*) (Fig. 1a). eEF1A is the most abundant protein in the yeast cytosol [24]. eEF2 is seven times less abundant than eEF1A [24]. Western blot methods may lack the dynamic range or sensitivity to detect a small or even moderate decline when abundant proteins are being assessed. For this reason, we focused on the least abundant protein, eEF2, and found that eEF2 levels declined when Emw1/TTC27 action was limited (Fig. 2a-f). Additionally, eEF2 is encoded by two genes, *EFT1* and *EFT2*, both genes encode identical proteins, specifically *EFT1* produces 30%, and *EFT2* produces 70% of the total transcript [25], so we could also begin with a reduced amount of eEF2 by using the *eft2Δ* background (as the only source of eEF2 is now the *EFT1* gene). This approach was effective, when Emw1 action was reduced we could detect a modest reduction in eEF2 levels, but a much greater decline was noted if we repeated the experiment in the *eft2Δ* (compare Fig. 2a-d). We did not see a reduction in eEF1A when we compromised Emw1 action, even in the *tef1Δ* background that lacks one of the genes encoding eEF1A (Fig. S2a). This wasn’t a surprise given that levels of eEF1A in the *tef1Δ* are still very high. Specifically, the amount of eEF1A in this background would be ten times higher than the amount of eEF2 in the *eft2Δ*, as calculated using the median abundance values of these proteins determined in a recent study [24]. Consequently, the folded pool of eEF1A may remain substantially in excess of functional threshold, so even if there is a decline in levels, the decline maybe not be evident in a quantity‐based assay. Furthermore, when Emw1 action is limited, we did not see an effect on eEF1A levels in the *hgh1Δ* or the *eft1Δhgh1Δ* background (Fig. S2b and Fig. S2c). This was not surprising as an interaction between Hgh1 and eEF1A has not been reported, implying that Hgh1 does not play a role in eEF1A maturation. In contrast, five separate studies have detected an interaction between eEF2 and Hgh1 [3, 4, 10, 33, 34]. Consequently, it is not surprising that a detrimental effect on eEF2 stability is detected in the *hgh1Δ* [3, 4].

## Discussion

Emw1/TTC27 is a hitherto poorly characterized protein, despite the corresponding gene being essential in yeast. Not surprisingly, the human orthologue (TTC27) is one of the ca. 2,000 genes identified as part of a core set of human genes that are required for context-free cell viability [35]. An in vivo protein complementation assay (PCA) reveals that Emw1/TTC27 physically interacts with the trGTPase proteins eEF2 (Eft2 in *S.cerevisiae*) and eEF1A (Tef1 in *S.cerevisiae*) (Fig. 1a). The read-out for interaction is growth on media containing methotrexate; initial observation of growth rates suggests that Emw1/TTC27 binding affinity for eEF1A is greater than that for eEF2 (Fig. 1a). DNA encoding the complementary fragments of mDHFR are fused to the 3’ ends of the chromosomal copies of genes, so expression is driven by the native promoter, and proteins will accumulate within the cell at native levels [13]. Accordingly, it is more likely that growth rates reflect the relative abundance of the two trGTPases as opposed to avidity of interaction, because levels of eEF1A are approximately seven times higher than levels of eEF2 [24].

In prior studies, the sole evidence used to classify a protein as a chaperone substrate was a decline in its steady-state level following inhibition of the chaperone. For instance, an interaction between Raf kinase and the chaperone Hsp90, plus levels of Raf declining when Hsp90 action is inhibited - were sufficient as evidence that Hsp90 is a chaperone for this kinase [36]. Indeed there are numerous studies where this loss of stability has been the index used to identify chaperone clients on the proteome-wide scale, for example identifying substrates of Hsp90 [37–39], Hsp60 [40], Hsp70 [41], and the prokaryotic chaperone GroE [42]. Proteins whose steady-state levels drop after selective inhibition of a chaperone can be classified as substrates of that chaperone**.** Additionally, when it is reported that a protein is a substrate for a specific chaperone, a physical interaction between the chaperone and substrate is always shown. Both of these conditions are met for the eEF2/Emw1 client/chaperone pairing, steady state levels of eEF2 decline when Emw1/TTC27 activity is limited (Fig. 2a-f) plus eEF2 and TTC27 physically interact (Fig. 1a). Accordingly, we claim that Emw1/TTC27 is a chaperone for eEF2.

Not surprisingly the interaction between the *emw1*^*ts*^ and eEF2 is far weaker than the interaction between wild-type Emw1 and eEF2 (Fig. 1d). This is true at the permissive temperature for the *emw1*^*ts*^ mutant, in agreement with our data demonstrating the hypomorphic nature of the *emw1*^*ts*^ mutant, specifically the decline in eEF2 levels (Fig. 2-f) and the severely compromised growth rate at permissive temperature (Fig. 3c and d).

eEF2 is the substrate of numerous chaperones including TRiC, Hsp90, and the recently discovered Hgh1. Compromising the action of Hsp90, or the Hsp90 co-chaperone Cns1, or the Hsp90 co-chaperone Cpr7, or Hgh1—all lead to a decrease in levels of eEF2 [3, 4, 43]. A similar effect is seen when Emw1/TTC27 is compromised, levels of eEF2 decline dramatically in *emw1*^*ts*^*eft2Δ* mutants relative to *EMW1eft2Δ* (Fig. 2c and d). The lower levels of eEF2 in *emw1*^*ts*^ cells results in growth phenotypes at 25^0^C or 30^0^C, while further reduction of the levels in *eft2Δ* cells is making translation elongation rates growth limiting, so its not surprising that compromising eEF2 levels still further in the *emw1*^*ts*^*eft2Δhgh1* is synthetic lethal at semi-nonpermissive temperature (Fig. 4b, sector 5). These robust synthetic defects, associated with combination of mutants that limit steady-state levels of eEF2, have been observed previously. Specifically, recent work from the Johnson lab reveal a severe limitation of growth in Hsp90 re-opening mutants in an *hgh1Δ* background [43].

Many Hsp90 co-chaperones, like Cns1 and HOP1, bind to Hsp90. Specifically, the interactions are between co-chaperone TPR domains and the Hsp90 C -terminal EEVD sequence. Even though there is limited amino acid similarity between TPR domains, alignment of the TPR domains known to bind Hsp90 displays a high degree of structural similarity, featuring five key residues that bind to the EEVD at the C terminus of Hsp90 [44]. Four of these residues are absent in Emw1/TTC27, one section with putative EEVD-binding residues is completely missing (Supplementary Fig. S3a). Sequence alignments can miss amino acid residues that are topologically conserved, but structural alignments between TPR domains of HOP and Emw1 indicated lack of conservation of these four Hsp90-binding residues (Fig. S3c), in contrast to the expected topological conservation of residues between two TPR domains known to bind Hsp90 (Fig. S3b). This poor alignment of Emw1/TTC27 TPR with TPR domains known to bind Hsp90, suggest there is no direct interaction between TPR domains of Emw1/TTC27 and Hsp90. In agreement with this, none of the numerous *S.cerevisiae* Hsp90-interactome analyses identify an interaction between these two proteins. We generated a recombinant vector that expressed a protein composed of (C mDHFR) fused to the N terminus of Hsc82 (one of the two Hsp90 paralogs of *S.cerevisiae*). This fusion did not interact with Emw1-(N mDHFR) (Fig. S4). Accordingly, despite the presence of TPR domains, it is unlikely that Emw1/TTC27 is a co-chaperone of Hsp90, in contrast to Cns1.

The soluble N terminal GTPase domain of eEF2 does not interact with Emw1/TTC27 (Fig. 7a). An in vivo test for interaction between Emw1/TTC27 and the C terminal fragment of eEF2 is not possible as this domain can’t be expressed in soluble form [4]. We have suggested that Emw1/TTC27 could interact with this C terminal domain, owing to a yeast two hybrid screen identifying the C terminal fragment of the splicing factor EFTUD2 (a close homolog of eEF2) as an interacting partner of TTC27. This is not sufficient to claim that this is the same binding site for eEF2. Given the lack of solubility of the eEF2 C terminal domain, testing the idea that Emw1/TTC27 interacts with this domain is technically challenging, and can only be addressed using expression of the intact eEF2. There is the additional complication that interaction between Emw1/TTC27 and eEF2 may only occur in a transient co-translational manner. These obstacles could be overcome by site-specific incorporation of unnatural photo reactive amino acids followed by photo crosslinking in vivo, and peptide mass fingerprinting—an approach that has been used to good effect in a recent study that identified the sites of interaction between Hsp90 and its clients [45].

Cellular levels of eEF2 are ca. nineteen times higher than levels of Hgh1 [24], which is not surprising as Hgh1 is required only during one stage during the lifetime of eEF2, specifically a transient interaction with domain III during eEF2 biosynthesis [4]. Cellular levels of eEF2 are ca. twenty-five times higher than levels of Emw1/TTC27 [24], in agreement with our data that suggest a similar type of transient co-translational interaction. Emw1 interacting with eEF2 during translation is consistent with the enrichment of ribosomal proteins found when protein–protein interaction data sets are interrogated via the Saccharomyces Genome Database. Specifically, of the fifty proteins found in complexes containing Emw1, thirteen are ribosomal polypeptides [46]. A similar observation is made when identifying the proteins in Hgh1 pull-downs [4].

**Fig. 10:**
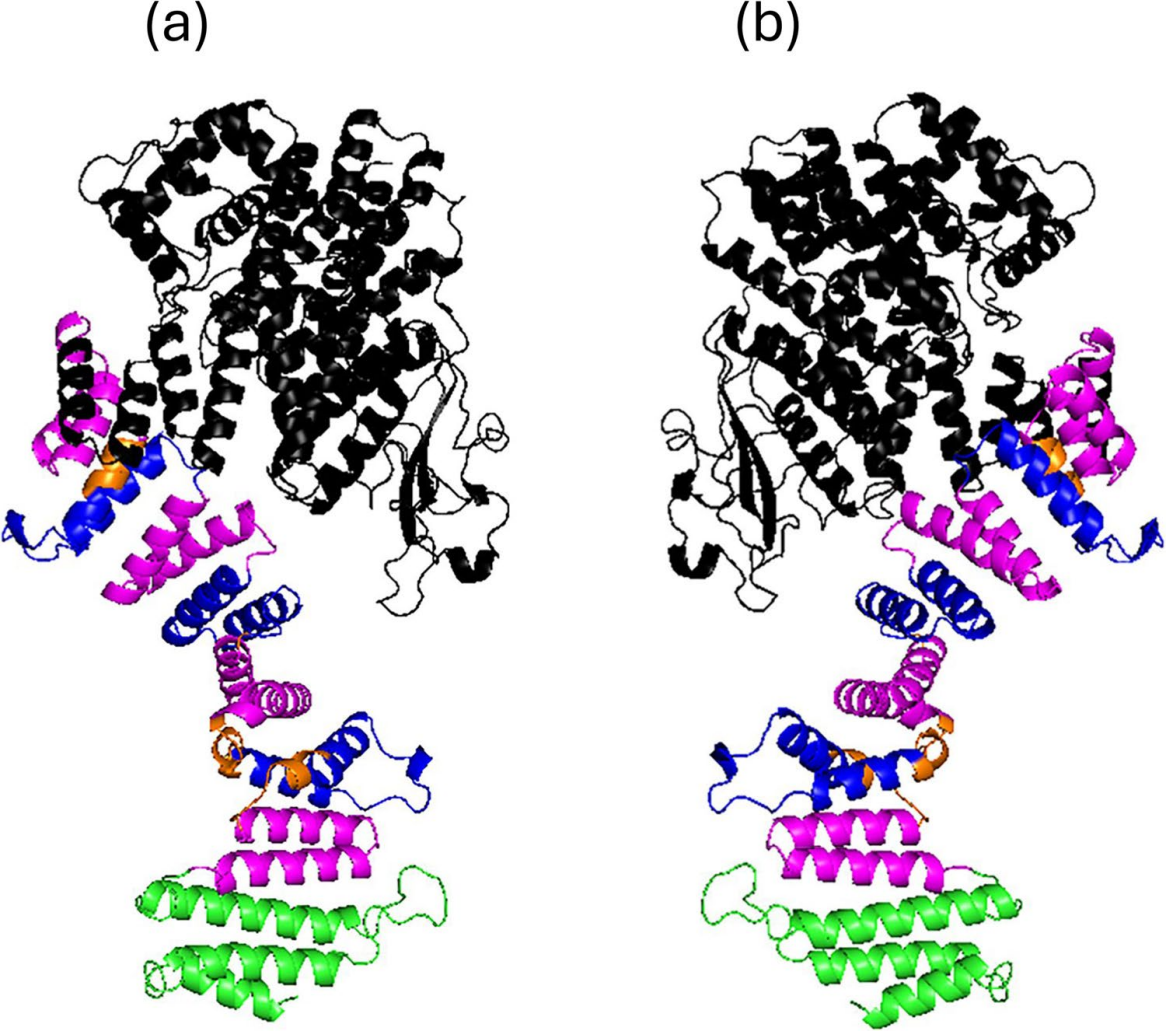
Emw1/TTC27 structure predicted by Alphafold [47]. Predicted structure of the *S.cerevisiae* orthologue (Emw1) is presented here (accession no. QHB11081.1). N terminal domain in black, C terminal domain in green. The domains are connected by a solenoid composed of six tetratricopeptide repeats (TPR) indicated in alternating blue and magenta (**b**) is a 180^0^ rotation of (**a**)

Focusing on the yeast orthologs, we used AlphaFold to predict the structure of the complex formed between Emw1/TTC27 and eEF2 [47]. To begin with, Alphafold predicts the structure of Emw1 at high confidence, with a predicted template modeling (pTM) score of 0.79 (values of 0.5 or greater are high quality predictions). There are six TPR motifs clustered in the C-terminal region, forming a long solenoid that would enable interaction with multiple binding partners or multiple contacts with different regions of the same binding partners. The TPR domains are arranged in a continuous array like the three TPR domains in the co-chaperone Cns1, in contrast to the three TPR domains distributed across the polypeptide sequence of HOP [48, 49]. Formation of a large cavity is predicted between the N terminal domain and the long solenoid (Fig. 10). Using AlphaFold3 to predict the structure of the complex formed when Emw1 binds to eEF2 was not successful [50]. The interface template modeling (ipTM) score measures the accuracy of the predicted relative positions of the subunits within a complex; a value > 0.8 represents a confident high-quality prediction, whereas a value < 0.6 suggest a failed prediction. The ipTM score for the predicted structure of an Emw1-eEF2 complex is 0.54 and falls within the zone of low confidence. A low confidence score may be due to predicting the structure of the complex based on an incorrect stoichiometry of 1:1. We have shown that an interaction can occur between two Emw1 polypeptides (Fig. 1a), suggesting that Emw1 may function as an oligomer. Alternatively, AlphaFold provides static models of protein–protein interaction, but it is not suited for predicting co-translational folding dynamics. As such it may be a poor predictor of the structures formed between eEF2 folding intermediates and chaperones like Emw1/TTC27.

At this stage, we can only speculate as to why Emw1/TTC27 is essential and Hgh1 is non-essential. Aberrant folding intermediates of eEF2 may occur frequently, owing to its elevated rates of synthesis in tandem with its complex multi-domain structure. This imposes a significant burden on the protein folding machinery, necessitating the sequential action of many chaperones. There may be an absolute requirement for the action of some chaperones (like Emw1/TTC27), whereas others are not essential but are required for optimal eEF2 folding (like Hgh1). Though compromised Emw1 or loss of Hgh1 activity leads to a similar outcome, namely lower steady-state levels of eEF2, the fate of unstable eEF2 is different. Aggregates of eEF2 accumulate in the *hgh1Δ* [4], but in *emw1*^*ts*^ mutants misfolded eEF2 is pushed towards clearance, with eEF2 aggregates not detected (Fig. 5c). There are numerous instances where the fate of the same client protein is different depending upon which of its chaperones has been inhibited. Inhibition of Hsp70 leads to accumulation of polyQ expanded Androgen receptor aggregates [51], whereas inhibition of Hsp90 promotes degradation of misfolded forms of the same client [52]. Knockdown of an Hsp40 family member leads to accumulation of the RNA binding protein TDP-43 aggregates [53], whereas depleting Cdc37 (an Hsp90 co-chaperone) causes TDP-43 to undergo proteolytic clearance [54].

A different form of eEF2 misfolded state may accumulate in cells that lack Hgh1 in contrast to that which accumulates when Emw1 action is compromised. Lack of Hgh1 could lead to a high concentration of exposed hydrophobic surfaces, forming oligomers quickly, outrunning both the ability of other chaperones to bind them or the action of mechanisms that would degrade them. In contrast, the exposed surfaces of eEF2 that accumulate when Emw1 action is impaired, could be bound more effectively by other chaperones, maintaining the misfolded protein in a soluble, chaperone-bound state long enough to facilitate its disposal.

Overall, it is difficult to address the contrast in phenotype between *hgh1Δ* (viable) and *emw1Δ* (lethal). This is because we do not know the identity of all clients for both chaperones. We have begun to address this by screening for binding partners for the human orthologue (TTC27), identifying RNA binding motif containing 12 (RBM12), the tumor suppressor tuberin (TSC2) and eukaryotic translation elongation factor 1 A, EEF1A1, a paralog of EEF1A (Fig. 9). The interaction between EEF1A and TTC27 is conserved as we also detect it between the yeast orthologous pair Tef1 and Emw1 (Fig. 1a). *S.cerevisiae* does not have TSC2 or RBM12 orthologs, so we can’t test conservation of interaction in these cases. It remains to be seen whether TSC2, RBM12 or EEF1A are *bona fide* clients of Emw1/TTC27. To begin with, it should be determined whether levels of these putative clients decline during TTC27 knockdown in a mammalian cell culture model.

Elongation factor eEF2 demands a major input from cellular chaperone machinery owing to its multi domain structure, the metastability of GTPase domains, and its high rates of synthesis. Indeed, overexpression of eEF2 has a growth limiting effect in mutants where activity of the chaperones Cpr7 and Cns1 are compromised [3, 43]. Not surprisingly, it has been shown that cells keep levels of eEF2 constant [55]. We suggest that Emw1/TTC27 is a new addition to the complement of chaperones required for eEF2 folding, its role as a chaperone not being revealed previously owing to its low abundance and transient interaction with its client. A transient co-translational interaction may explain why proteome-wide yeast two hybrid screens [56, 57] and our two hybrid tests failed to detect an interaction between Emw1/TTC27 and eEF2. This failure is likely due to two hybrid screens—that exploit re-formation of the GAL4 transcription factor—detecting interactions between fully folded proteins that have transited into the nucleus. We did identify binding targets to human TTC27 using a two hybrid screen, but these interaction partners were truncated or partial fragments of proteins and it is well-established that non-native or mutant forms of chaperone clients can be unstable and necessitate a greater requirement for chaperone interaction in order to maintain stability of the client [58]. Emw1/TTC27 now joins a fleet of recently discovered chaperones including Hgh1, Zpr1, Aim29 and Chp1, all of which have several features in common, namely their low abundance and transient co-translational interaction with their trGTPase elongation factor clients, EEF1A or eEF2 [3, 4, 59–61].

## Materials and methods

### Yeast strains and genotypes

#### Yeast cell culture

Strains were grown in either rich medium (YPD) or synthetic minimal medium (SD), with appropriate supplements or selective antibiotics for plasmid maintenance [63].

Haploid strains listed in Table 1 that express proteins tagged with either the C or N—terminal domain of murine dihydrofolate reductase were obtained from Dharmacon (via Horizon Discovery, United Kingdom).

**Table 1:** Yeast strains and genotypes used in this study

Strain	Genotype	Source
PJ69-4a	*MATa trp1-901 leu2-3,112 ura3-52 his3-200 gal4Δ gal80Δ lys2::GAL1-HIS3 GAL2-ADE2 met2::GAL7-lacZ*	[62]
PJ69-4α	*MATα trp1-901 leu2-3,112 ura3-52 his3-200 gal4Δ gal80Δ lys2::GAL1-HIS GAL2-ADE2 met2::GAL7-lacZ*	[62]
MY30	*MATα trp1-901 leu2-3,112 ura3-52 his3-200 gal4Δ gal80Δ lys2::GAL1-HIS GAL2-ADE2 met2::GAL7-lacZ;* bearing pGBDU-TTC27 (*GAL4BD-TTC27*)	This study
BY4741	*MAT****a**** his3Δ leu2Δ met15Δ ura3Δ*	EUROSCARF
BY4742	*MATα his3Δ leu2Δ lys2Δ ura3Δ*	EUROSCARF
*MAT* a Emw1	*MAT****a****; his3Δ; leu2Δ; met15Δ; ura3Δ;YNL313C-F*[1,2]*::nat1*	Dharmacon™
*MAT* a Sec6	*MAT****a**** his3Δ leu2Δ met15Δ ura3Δ YIL068C-F*[1,2]*::nat1*	Dharmacon™
*MAT* α Sec5	*MATα his3Δ leu2Δ; lys2Δ ura3Δ YDR166C-F*[3]*::hph*	Dharmacon™
*MAT* α Emw1	*MATα his3Δ leu2Δ lys2Δ ura3Δ YNL313C-F*[3]*::hph*	Dharmacon™
*MAT* α Eft2	*MATα his3Δ leu2Δ lys2Δ ura3Δ YDR385W-F*[3]*::hph*	Dharmacon™
*MAT* α Tef1	*MATα his3Δ leu2Δ lys2Δ ura3Δ YPR080W-F*[3]*::hph*	Dharmacon™
*MAT* α Rho1	*MATα his3Δ leu2Δ lys2Δ ura3Δ YPR165W-F*[3]*::hph*	Dharmacon™
*MAT* α Ras1	*MATα his3Δ leu2Δ lys2Δ ura3Δ YOR101W-F*[3]*::hph*	Dharmacon™
*MAT* α Ypt1	*MATα his3Δ leu2Δ lys2Δ ura3Δ YFL038C-F*[3]*::hph*	Dharmacon™
*MAT* α Cin4	*MATα his3Δ leu2Δ lys2Δ ura3Δ YMR138W-F*[3]*::hph*	Dharmacon™
*MAT* α Gsp1	*MATα his3Δ leu2Δ lys2Δ ura3Δ YLR293C-F*[3]*::hph*	Dharmacon™
*MAT* α Arf1	*MATα his3Δ leu2Δ lys2Δ ura3Δ YDL192W-F*[3]*::hph*	Dharmacon™
SA2	*MATα his3Δ1 leu2Δ0 ura3Δ0 lys2Δ0 MET15* *emw1::kanMX4,* bearing *pY33T-E plasmid (EMW1/URA3)* and pY111T-Emw1 (*EMW1/LEU2)*	[14]
SA2i	*MATα his3Δ1 leu2Δ0 ura3Δ0 lys2Δ0 MET15* *emw1::kanMX4,* bearing *pY33T-E plasmid (EMW1/URA3)* and pY111T-emw1^ts^ (*emw1-1*^*ts*^*/LEU2)*	[14]
SP0	*MATα his3Δ1 leu2Δ0 ura3Δ0 lys2Δ0 MET15* *emw1::kanMX4,* bearing pY111T-EMW1 plasmid *(EMW1/LEU2)*	[14]
SP1	*MATα his3Δ1 leu2Δ0 ura3Δ0 lys2Δ0 MET15* *emw1::kanMX4,* bearing pY111T-emw1^ts^ plasmid* (emw1*^*ts*^*/LEU2)*	[14]
MY53	*MATα his3Δ1 leu2Δ0 ura3Δ0 lys2Δ0 MET15* *emw1::kanMX4 eft2::hph,* bearing *pY33T-E plasmid (EMW1/URA3)* and pY111T-Emw1 (*EMW1/LEU2)*	This study
MY54	*MATα his3Δ1 leu2Δ0 ura3Δ0 lys2Δ0 MET15* *emw1::kanMX4 eft2::hph,* bearing *pY33T-E plasmid (EMW1/URA3)* and pY111T-emw1^ts^ (*emw1-1*^*ts*^*/LEU2)*	This study
MY68	*MATα his3Δ1 leu2Δ0 ura3Δ0 lys2Δ0 MET15 emw1::kanMX4 eft2::hph,* bearing pY111T-EMW1 plasmid *(EMW1/LEU2)*	This study
MY69	*MATα his3Δ1 leu2Δ0 ura3Δ0 lys2Δ0 MET15* *emw1::kanMX4 eft2::hph,* bearing pY111T-emw1^ts^ plasmid* (emw1*^*ts*^*/LEU2*	This study
MY50	*MATα his3Δ1 leu2Δ0 ura3Δ0 lys2Δ0 MET15 emw1::kanMX4 hgh1::nat1,* bearing *pY33T-E plasmid (EMW1/URA3)* and pY111T-Emw1 (*EMW1/LEU2)*	This study
MY51	*MATα his3Δ1 leu2Δ0 ura3Δ0 lys2Δ0 MET15 emw1::kanMX4 hgh1::nat1,* bearing *pY33T-E plasmid (EMW1/URA3)* and pY111T-emw1^ts^ (*emw1-1*^*ts*^*/LEU2)*	This study
MY66	*MATα his3Δ1 leu2Δ0 ura3Δ0 lys2Δ0 MET15* *emw1::kanMX4 hgh1::nat1,* bearing pY111T-EMW1 plasmid *(EMW1/LEU2)*	This study
MY67	*MATα his3Δ1 leu2Δ0 ura3Δ0 lys2Δ0 MET15* *emw1::kanMX4 hgh1::nat1,* bearing pY111T-emw1^ts^ plasmid* (emw1*^*ts*^*/LEU2*	This study
Y10000	*MATα his3Δ1 leu2Δ0 ura3Δ0 lys2Δ0 MET15*	EUROSCARF
Y14817	*MATα his3Δ1 leu2Δ0 ura3Δ0 lys2Δ0 MET15 hgh1::kanMX4*	EUROSCARF
MY59	*MATα his3Δ1 leu2Δ0 ura3Δ0 lys2Δ0 MET15 emw1::kanMX4 hgh1::nat1 eft2::hph,* bearing *pY33T-E plasmid (EMW1/URA3)* and pY111T-Emw1 (*EMW1/LEU2)*	This study
MY60	*MATα his3Δ1 leu2Δ0 ura3Δ0 lys2Δ0 MET15 emw1::kanMX4 hgh1::nat1 eft2::hph,* bearing *pY33T-E plasmid (EMW1/URA3)* and pY111T-emw1^ts^ (*emw1-1*^*ts*^*/LEU2)*	This study
MY81	*MATα his3Δ1 leu2Δ0 ura3Δ0 lys2Δ0 MET15 emw1::kanMX4 tef1::hph,* bearing pY111T-EMW1 plasmid* (EMW1/LEU2)*	This study
MY82	*MATα his3Δ1 leu2Δ0 ura3Δ0 lys2Δ0 MET15 emw1::kanMX4 tef1::hph,* bearing pY111T-emw1^ts^ (*emw1-1*^*ts*^*/LEU2)*	This study
MY83	*MATα his3Δ1 leu2Δ0 ura3Δ0 lys2Δ0 MET15 emw1::kanMX4 tef1::hph hgh1::nat1* bearing pY111T-EMW1 plasmid* (EMW1/LEU2)*	This study
MY84	*MATα his3Δ1 leu2Δ0 ura3Δ0 lys2Δ0 MET15 emw1::kanMX4 tef1::hph hgh1::nat1* bearing pY111T-emw1^ts^ (*emw1-1*^*ts*^*/LEU2)*	This study

*EUROSCARF – European *Saccharomyces cerevisiae* archive for functional analysis

*F[1,2]* is the N terminal domain of mDHFR, amino acids 1–105 bearing mutations L22F F31S. *F[3]* corresponds to amino acids 106–186 of mDHFR [13]. The L22F F31S substitutions result in a reconstituted mDHFR that is insensitive to methotrexate [13]

Deletion of *EFT2* or *HGH1* was performed by replacing the ORF with *hph* (conferring resistance to hygromycin B) or *nat* (conferring resistance to nourseothricin) via one-step gene replacement, as previously described [64]. Integration at the correct locus was confirmed by diagnostic PCR.

### Protein extraction and detection of proteins

Cultures were grown to exponential phase and protein was extracted as described previously [65]. Proteins were resolved via SDS-PAGE using Any kD™ mini-PROTEAN® TGX™ precast gels (BIO-RAD, USA) and Western blots were probed for: eEF2 using a polyclonal anti-eEF2 cat.no. ARP58457_P050 (Aviva Systems Biology, USA), or anti-EEF1A cat.no ARP48166_T100 or Sba1 as loading control using a polyclonal anti-Sba1 [66]. For fractionation, protein lysates were split into soluble and insoluble fractions as previously described [4].

### Sensitivity to drugs

Exponentially growing cultures were adjusted to equal cell density (1 × 10^7^ cells/ml) and four successive five-fold serial dilutions were spotted on YPD, containing the concentration of drug indicated in the figure legend.

### Yeast two-hybrid screening

The cDNA fragment encoding human TTC27 (accession number NP_060205.3) was subcloned into a *URA3* episomal yeast expression plasmid pGBD-C1 [62] in-frame to the GAL4 DNA binding domain. This recombinant was transformed into strain PJ69-4α (Table 1). For screening of interaction partners, the Human Leukocyte Matchmaker™ cDNA Library was used (Takara, Japan). Preparation of all media and reagents and the execution of the two hybrid screen was performed according to the Matchmaker GAL4 Two-Hybrid System 3 protocol (Takara, Japan) using strain PJ69-4a expressing the TTC27-BD fusion as host, into which was transformed the Matchmaker™ cDNA Library, selecting transformants that express interacting partners of TTC27 on SD media lacking uracil, leucine and histidine. Interacting partners were re-tested by transforming the isolated cDNA-AD fusion plasmids into PJ69-4a and mating to the PJ69-4α expressing the TTC27-BD fusion [65].

## Supplementary Information

Below is the link to the electronic supplementary material.

Supplementary file1 (PDF 954 KB)

## Data Availability

Not applicable (no large datasets).
