# Supplementary material for: Emw1/TTC27 is a chaperone required for folding of the eukaryotic elongation factor 2
Source: Cell Mol Life Sci. 2026 Mar 10;83(1):192. doi: 10.1007/s00018-026-06154-9 (PMC13049181; doi:10.1007/s00018-026-06154-9)
Supplement: Supplementary file 1 — Supplementary file1 (PDF 954 KB) [file 18_2026_6154_MOESM1_ESM.pdf]

# Supplementary information

## Emw1/TTC27 is a chaperone required for folding of the eukaryotic Elongation Factor 2

Mengqi Yang[1,2], Ruixin Li[1], Anna I. Mikolajczak[1,3], Vanessa A. Wright[1], Mahnoor Hassan[1,], Cara K. Vaughan[4], Thomas A.K. Prescott[5], Jennifer A. Heritz[6,7,8], Mehdi Mollapour[6] and Barry Panaretou[1]*

[1]Institute of Pharmaceutical Science, School of Cancer and Pharmaceutical Sciences, King's College London, Franklin-Wilkins Building, 150 Stamford Street, London SE1 9NH, UK
[2]current address RNA Therapeutics Institute UMass Chan Medical School, Albert Sherman Center (AS4-2046), 368 Plantation Street, Worcester, MA 01605, USA
[3]current address Experimental Histopathology, The Francis Crick Institute, 1 Midland Road, NW1 1AT, London, UK.
[4]Department of Molecular Biosciences, University of Texas at Austin, Austin, TX, USA
[5]Royal Botanic Gardens, Kew, Richmond TW9 3AB, Surrey, UK
[6]Department of Urology, SUNY Upstate Medical University Syracuse, NY 13210, USA
[7]Department of Biochemistry and Molecular Biology, SUNY Upstate Medical University Syracuse NY 13210, USA
[8]Upstate Cancer Centre, SUNY Upstate Medical University Syracuse, NY 13210, USA

*Correspondence: barry.panaretou@kcl.ac.uk

(a)

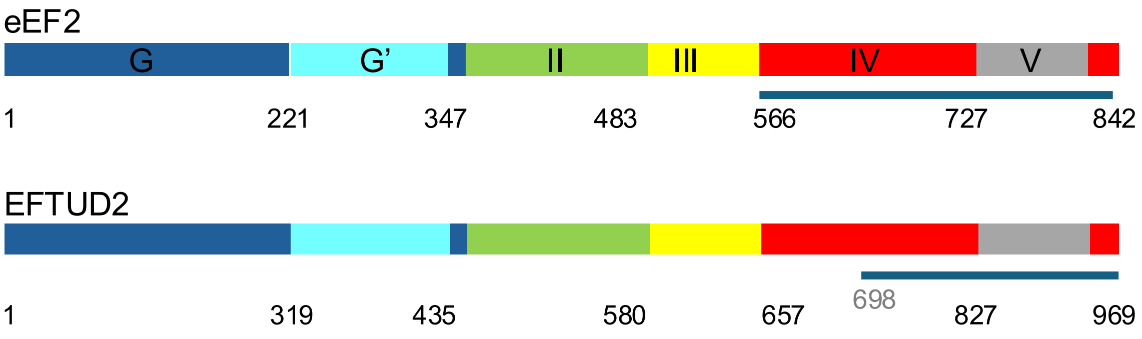


(b)

```
Eft2(IV-V)          TVESESSQTALSKSPNKHNRIYLKAEPIDEEVSLAIENGIINPRDDFKARARIMADDYGW      60
EFTUD2(272,Y2H)     ------------------------------------ENEVVQITWNRKKLGEFFQTKYDW      24
                                                        ** :::   : *  ..::  .*.*

Eft2(IV-V)          DVTDARKIWCFGPDGNGPNLVIDQTKAV----QYLHEIKDSVVAAFQWATKEGPIFGEEM      116
EFTUD2(272,Y2H)     DLLAARSIWAFGPDATGPNILVDDTLPSEVDKALLGSVKDSIVQGFQWGTREGPLCDELI      84
                    *:  **.**.****..***:::*:*         * .:***:* .***.*:***: .* :

Eft2(IV-V)          RSVRVNILDVTLHADAIHRGGGQIIPTMRRATYAGFLLADPKIQEPVFLVEIQCPEQAVG      176
EFTUD2(272,Y2H)     RNVKFKILDAVVAQEPLHRGGGQIIPTARRVVYSAFLMATPRLMEPYYFVEVQAPADCVS      144
                    *.*:.:***..:  : :********** **..*:.**:* *:: ** ::**:*.* :.*.

Eft2(IV-V)          GIYSVLNKKRGQVVSEEQRPGTPLFTVKAYLPVNESFGFTGELRQATGGQAFPQMVFDHW      236
EFTUD2(272,Y2H)     AVYTVLARRRGHVTQDAPIPGSPLYTIKAFIPAIDSFGFETDLRTHTQGQAFSLSVFHHW      204
                    .:*:** ::**:*..:   **:**:*:**::*. :****  :**  * ****   **.**

Eft2(IV-V)          STLGSDPLDPTSK------------AGEIVLAARKRHGMKEEVPGWQEYYDKL-------      277
EFTUD2(272,Y2H)     QIVPGDPLDKSIVIRPLEPQPAPHLAREFMIKTRRRKGLSEDVSISKFFDDPMLLELAKQ      264
                    . : .**** :              * *::: :*:*:*:.*:*   : : * :

Eft2(IV-V)          --------   277
EFTUD2(272,Y2H)     DVVLNYPM   272
```

**Fig. S1 Similarity between the C terminal domains of EFTUD2 and eEF2**
(a) schematic showing the similar domain structure of both proteins, the grey bars indicating the fragment of sequence used in the alignment for (b). The grey bar for EFTUD2 also represented the fragment of the protein identified from a two-hybrid screen (b) Alignment of the C terminal domain of human EFTUD2 (accession NP_004238.3) with the C -terminal domain of Eft2 (accession no. KZV12629, one of the two identical eEF2 orthologs expressed by *S.cerevisiae*).

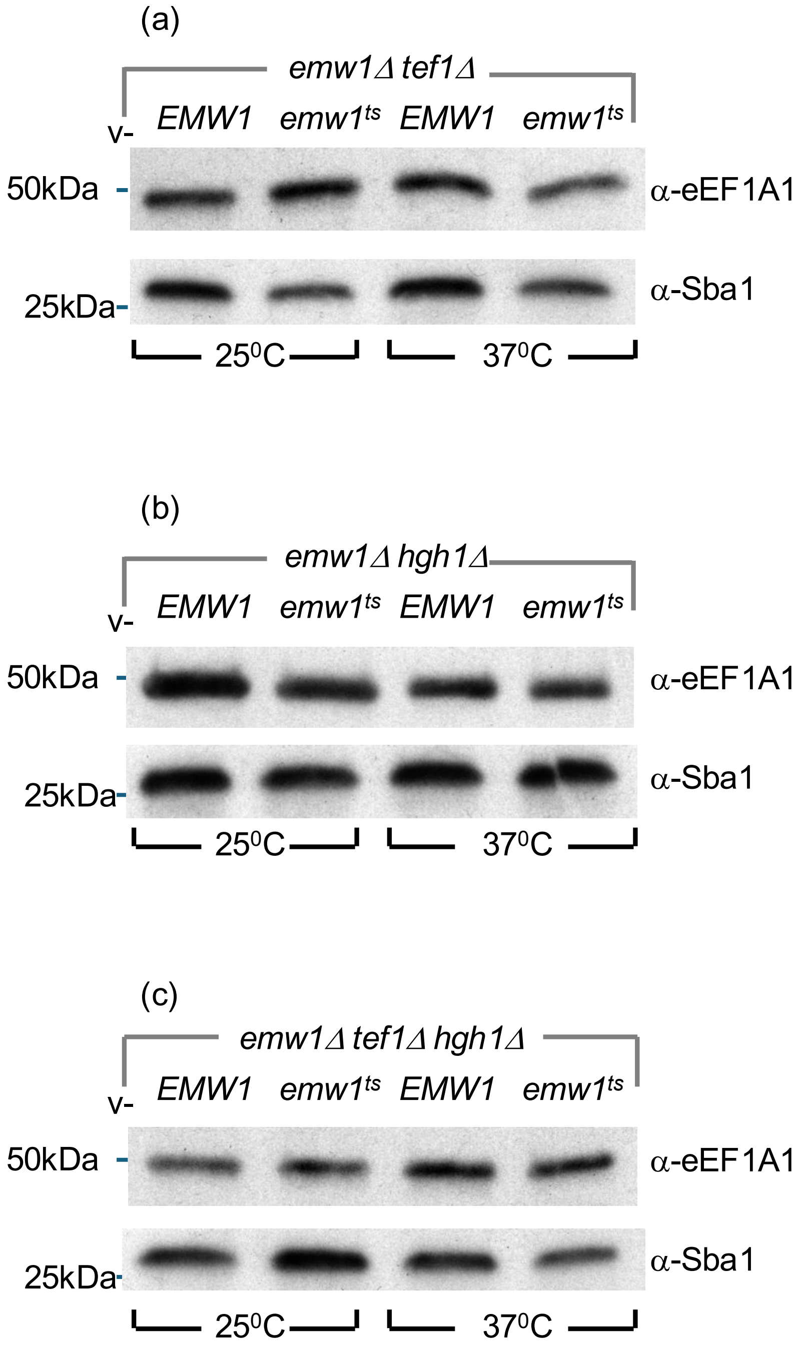


**Fig. S2 Levels of eukaryotic Elongation Factor 1A (eEF1A)**
*emw1Δ tef1Δ* (a), *emw1Δhgh1Δ* (b), and *emw1Δ tef1Δ hgh1Δ* (c), bearing a centromeric vector (v-) expressing either wild type *EMW1* or *emw1$^{ts}$* from the *EMW1* promoter. Cultures were incubated in YPD to exponential phase at 25°C, split into two equal aliquots which were either i) maintained at 25°C or ii) shifted to 37°C for four hours. Extracted proteins were resolved by SDS-PAGE and Western blots were probed with anti-eEF1A1 antisera or anti-Sba1 antisera (as loading control). Lanes were loaded with 4μg protein; molecular weight markers are indicated on the left.

**Supplementary Fig. S3**
(a) Primary sequence alignments.
Sequence alignment of TPR domains known to bind Hsp90, listed on the left-hand side are PDB accession numbers for empirically determined structures of the domains in complex with the peptide ligand derived from the C terminal EEVD of Hsp90. Arrows indicate conserved residues that bind EEVD [31]. These are aligned against the TPR domain of *S.cerevisiae* Emw1 (TPRlong: all six TPR motifs in Emw1).
Alignment by structural overlay.
(b) Overlays of Hsp90-binding TPR domains of HOP and PP5
TPR2A and TPR3B of HOP (green, residues 272-380) overlaid with TPR domain of PP5 (red, residues 28-129). All five Hsp90-binding residues of the HOP TPR domain (blue text) are topologically conserved in the Hsp90-bindiong TPR domain of PP5 (red text).
(c) Overlays of Hsp90-binding TPR domains of HOP and the TPR domain of Emw1.
TPR2A and TPR3B of HOP (green, residues 272-380) overlaid with TPR domain of Emw1 (magenta, residues 563-825). Four of the five Hsp90-binding residues of the HOP TPR domain (blue text) are not topologically conserved in the TPR domain of Emw1 (magenta text).
Accession numbers of sequences used HOP (NP_001269581.1), PP5 (NP_006238.1), Emw1 (NP_014086.1).

# Fig. S3a

(a)

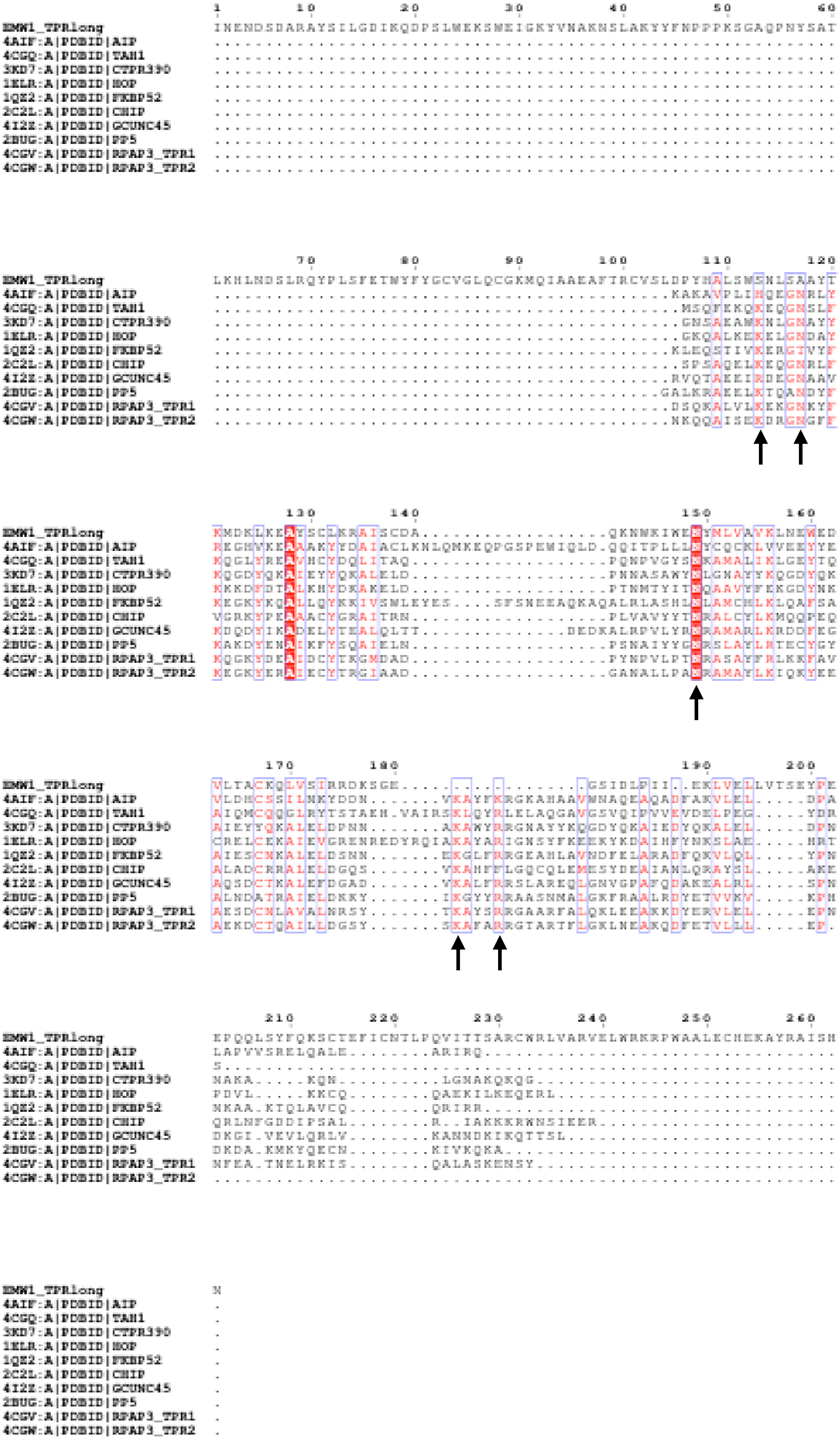
EMW1_TPRlong
4AIF:A|PDBID|AIP
4CGQ:A|PDBID|TAH1
3KD7:A|PDBID|CTPR390
1ELR:A|PDBID|HOP
1QZ2:A|PDBID|FKBP52
2C2L:A|PDBID|CHIP
4I2Z:A|PDBID|GCUNC45
2BUG:A|PDBID|PP5
4CGV:A|PDBID|RPAP3_TPR1
4CGW:A|PDBID|RPAP3_TPR2

Fig. S3

(b)

HOP/PP5 TPR overlay

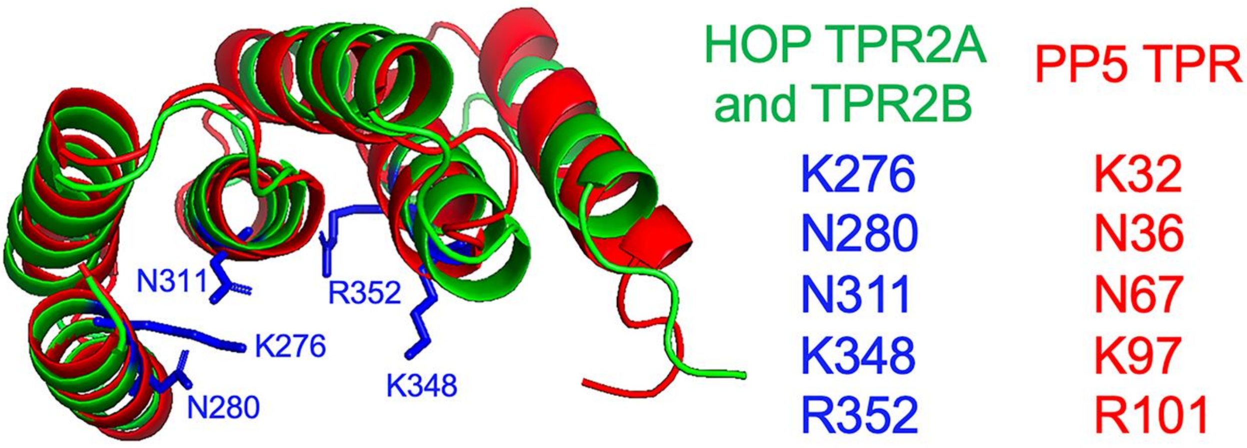


| HOP TPR2A and TPR2B | PP5 TPR |
|---|---|
| K276 | K32 |
| N280 | N36 |
| N311 | N67 |
| K348 | K97 |
| R352 | R101 |

(c)

HOP/Emw1 TPR overlay

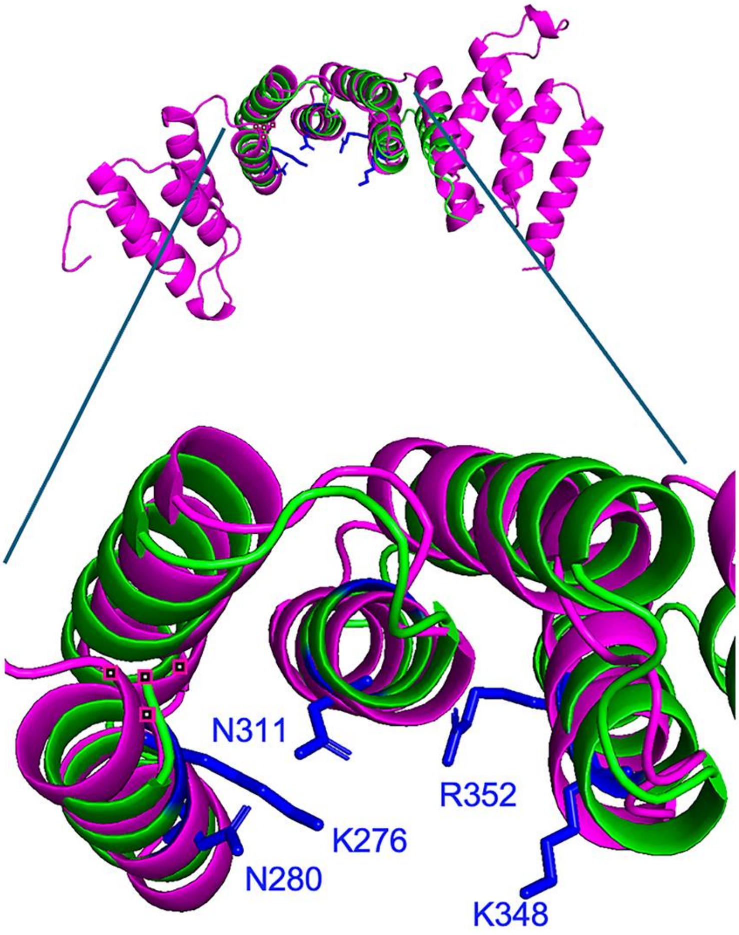


| HOP TPR2A and TPR2B | Emw1 TPR |
|---|---|
| K276 | Y641 |
| N280 | C645 |
| N311 | N676 |
| K348 | I708 |
| R352 | N711 |

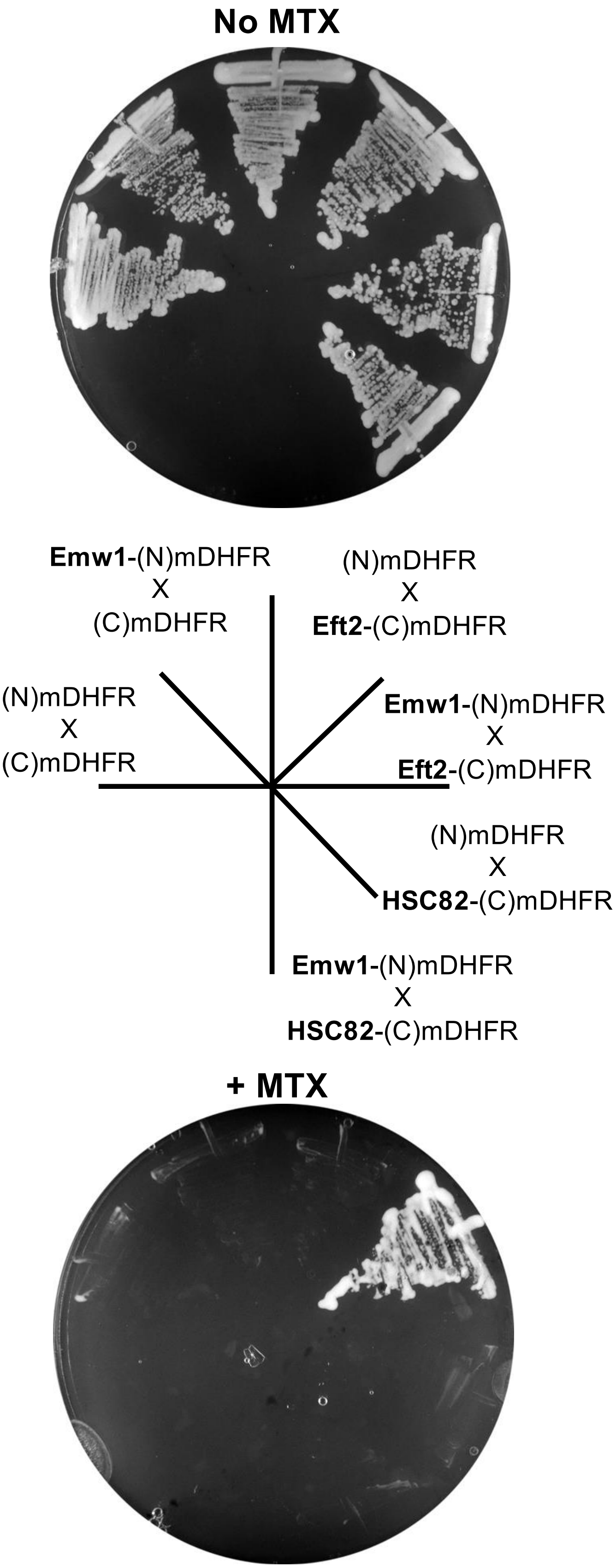


**Fig. S4. Interaction between Emw1 and Hsp90 is not detected by the protein complementation assay.** *S. cerevisiae* bearing episomal plasmids expressing the indicated pair-wise combinations of (N) mDHFR, (C)mDHFR, Emw1-(N)mDHFR, HSC82-(C)mDHFR and Eft2-(C)mDHFR, all under the control of the constitutively active promoter of *ADH1*. Cells were incubated at 30°C for 2 days on SD media lacking leucine and uracil (No MTX) and the same media also containing 100 μg/ml methotrexate (+MTX). Hsp90 encoded by *S.cerevisiae HSC82*.
